# Exercise training improves blood pressure reactivity to stress: a systematic review and meta-analysis

**DOI:** 10.1038/s41598-023-38041-9

**Published:** 2023-07-06

**Authors:** Igor M. Mariano, Ana Luiza Amaral, Paula A. B. Ribeiro, Guilherme Morais Puga

**Affiliations:** 1 Laboratory of Cardiorespiratory and Metabolic Physiology, Physical Education Department, Federal University of Uberlândia, Rua Benjamin Constant, 1286. Bairro: Aparecida, Uberlândia, MG 38400-678 Brazil; 2 Research Center of University of Montreal Hospital Centre, Montreal, QC H2X 0A9 Canada; 3 Centre de Médecine Comportemental de Montréal, CIUSSS-NIM, Montreal, QC H4J 1C5 Canada

**Keywords:** Hypertension, Risk factors, Hypertension

## Abstract

Blood pressure (BP) reactivity to stress is associated with cardiovascular events and the incidence of hypertension, therefore, tolerance to stressors is important for better management of cardiovascular risks. Exercise training is among the strategies that have been investigated as blunting the peak response to stressors, however, its efficacy is poorly explored. The aim was to explore the effects of exercise training (at least four weeks) on BP responses to stressor tasks in adults. A systematic review was performed in five electronic databases (MEDLINE, LILACS, EMBASE, SPORTDiscus, and PsycInfo). Twenty-three studies and one conference abstract was included in the qualitative analysis, totaling 1121 individuals, and k = 17 and 695 individuals in the meta-analysis. Favorable results (random-effects) for exercise training were found, with attenuated peak responses in systolic (standardized mean difference (SMD) = −0.34 [−0.56; −0.11], representing average reductions of 2.5 ± 3.6 mmHg) and null effects on diastolic BP (SMD = −0.20 [−0.54; 0.14], representing average reductions of 2.0 ± 3.5 mmHg). The analysis removing outliers’ studies improved the effects for diastolic (SMD = −0.21 [−0.38; −0.05]) but not systolic BP (SMD = −0.33 [−0.53; −0.13]). In conclusion, exercise training seems to lower stress-related BP reactivity, therefore has the potential to improve patients’ ability to better respond to stressful situations.

## Introduction

Modern life provides several stressful situations in which homeostasis is challenged^1^. Studies have shown that blood pressure (BP) alterations in response to stressors (i.e., BP reactivity) are associated, with the development of future cardiovascular events^2,3^, hypertension^4,5^, and decreased telomere length independent of resting BP^2^. There are indications that cardiovascular responses to stress are better predictors of left ventricular mass^6^ and incidence of hypertension^4,7^ than resting BP. As a result, assessing BP reactivity through simple laboratory tests could be a valuable tool for cardiovascular risk stratification.

Different stress protocols have been used in the literature. A literature review identified studies that involve physical stressors (e.g. physiological or environmental), mental stressors (e.g. emotional or cognitive), or a mix of both^3^. These stressors can trigger responses from different mechanisms that could explain the increase in BP levels^1,8,9^, such as (1) increased secretion levels of adrenaline/noradrenaline^1,10,11^ and cortisol^12–14^; (2) alterations in neural-network, such as salience network, default mode network, and executive control network^15,16^; and (3) responses of the autonomic system reducing vagal tone^17–20^.

Exercise training is one of the most prescribed non-pharmacological strategies to control high BP^21^ and therefore also important to be studied under stressful situations. Previous meta-analysis about the effect of a single session of aerobic exercises (i.e., acute exercise) on BP reactivity^22,23^ found attenuated peak BP responses, regardless of population, type of stressors, or study design characteristics. Moreover, a systematic review^24^ assessed the effects of exercise training (i.e., chronic exercise) and aerobic physical fitness on several cardiovascular markers and found blunted BP reactivity results, reiterating the importance of exercise to mitigate peak BP responses. However, there are still some inconsistencies in the literature, showing no effect of physical fitness^25^, and the influence of non-aerobic exercise training on BP reactivity to stressful situations is still poorly understood.

Therefore, the objective of the present study was to investigate the effects of chronic exercise training on BP reactivity in response to stressor tasks in adults. In addition, explore the influence of exercise characteristics (e.g., exercise mode), stress tests idiosyncrasies (e.g., type and number of stressors, and data presentation), and population characteristics (e.g., sex, age, and presence of hypertension) on BP reactivity after exercise training. Our hypothesis is that exercise training, attenuates BP reactivity to stress, reducing peak BP responses in these individuals, similar to the response already demonstrated after acute exercise^22,23^.

## Methods

This systematic review and meta-analysis was registered on the “PROSPERO” platform (CRD42020195700), had its protocol published on the “protocols.io” platform^26^, and followed PRISMA guidelines^27,28^.

### Eligibility criteria

Studies with the following characteristics were eligible: only interventional clinical trials in human adults (> 18 years) of both sexes; the intervention was exercise training for at least 4 weeks and a control group without exercise; the outcome of interest was BP reactivity (peak BP or BP variation from baseline) during laboratory stressor tasks (except treadmill cardiopulmonary test) after exercise training (including if it is a secondary data analysis); and studies in English, Portuguese, or Spanish with no publication dates limitation were included.

The exclusion criteria were literature reviews, meta-analysis, observational studies, studies with non-structured exercises such as relaxation, stretching, and breathing exercises (i.e. where intensity is objectively unmeasurable), studies that included patients after cardiovascular events (i.e. cardiovascular rehabilitation), and studies that did not measure BP during the stress tests.

### Search strategy

**Figure 1:**
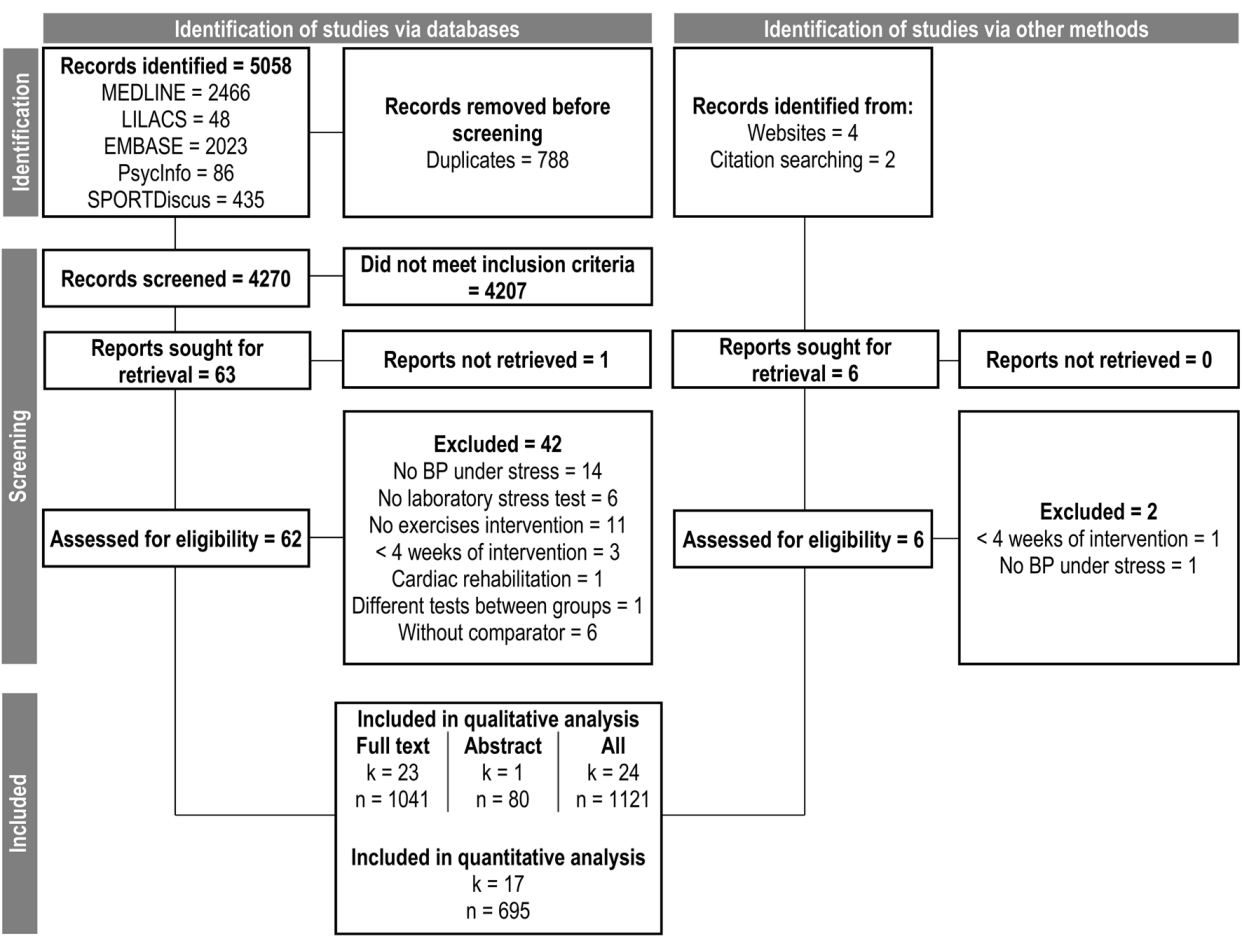
Flow diagram. *K* number of studies, *n* pooled sample size, *BP* blood pressure.

The searches were performed in five electronic databases (MEDLINE, LILACS, EMBASE, SPORTDiscus and PsycInfo), in the list of references of the main articles, and through manual search (“https://core.ac.uk/” and “https://scholar.google.com/”) until November 29th/2022. The flow diagram is shown in Fig. 1. When necessary, we contacted the authors requesting the missing information. The search was divided into three categories of terms: (1) Exercise, (2) Blood pressure; and (3) Stress tests. Within each category, the terms were separated by union operators (i.e., “OR”), and the categories were separated by parentheses and intersection operators (i.e., “AND”) in the following format:

(*Exercise OR “Exercise Therapy” OR “Physical activity” OR “Physical training” OR Aerobic OR Cycling OR Bicycle OR Treadmill OR “Cycle ergometer” OR Cyclergometer OR “Cycle-ergometer” OR Swimming OR Swim OR Running OR Run OR “Hand grip” OR “Hand-grip” OR Walking OR Walk OR “Weight training” OR “Weight-training” OR “Weight exercise” OR “Weight-exercise” OR “Resistance exercise” OR “Resistance training” OR Strength OR Pilates OR Yoga OR Ioga OR Taichi OR “Tai chi” OR “Tai-chi” OR Isometric OR Hiit OR Hit OR Siit OR Sit OR “High intensity” OR “Moderate intensity” OR ”Low intensity” OR “Combined training” OR “Combined exercise” OR “Concurrent training” OR “Concurrent exercise”) AND (“Arterial pressure” OR “Blood pressure” OR Diastolic OR Systolic) AND (“Reactivity” OR “Cold pressor” OR “Stroop” OR “Stress test” OR Psychosocial OR “Psychosocial test” OR “Psychosocial stress” OR “Psychosocial task “ OR “Stress task” OR “math task” OR “Speech task” OR Speech OR Math OR Arithmetic OR “Arithmetic test” OR “Arithmetic task”*)*.*

### Screening and data extraction process

During the entire process, the studies were evaluated in duplicate by independent reviewers (IMM and ALA) for screening, data extraction, and risk of bias assessment. The disagreements were resolved by consensus or by a third reviewer when necessary (GP). After the title and abstract screening phase, one of the reviewers standardized alphanumeric codes for all studies. Then, each reviewer independently filled out a datasheet detailing the characteristics of the studies, and the data were compared to assess agreement and identify errors. This datasheet included a general description (identification code, author, publication year, language, and study design), participants’ description (sexes, sample size, participants’ health condition, fitness status, age, hypertension status, and other comorbidities), exercise description (intensity, volume, frequency and exercise mode); stressor task description (stressor test, and BP measurement technique), and outcome measures (SBP and DBP reactivity) for intervention and comparator groups (sample sizes, data centrality and dispersion measures). In studies in which the data are presented only in graphs or figures without clear numerical representation, the data were extracted by the web-based software “WebPlotDigitizer” (analysis performed in 9 studies)^29–37^. When there was not enough data for quantitative analysis, the authors were contacted.

### Statistical analysis

Meta-analyses were performed using the “R” programming language through the packages “meta”^38^ and “metafor”^39^. Effects are described using standardized mean differences (SMD), since it allows a weighted understanding of the intervention effect size, in addition to being a more generalizable type of measure than the mean differences^40,41^. However, we also describe the mean differences in clinical units (i.e., mmHg). When necessary, data were transformed into mean and standard deviation. If presented in standard errors, we used the following formula “Standard deviation = standard error * √sample size”, and when presented as median and interquartile ranges, we used the methods previously described^42,43^.

Comparisons were made from BP reactivity data (peak BP or change in BP from baseline, both transformed into SMD for analysis) after the exercise training phase compared to a non-exercise group. In studies that presented multiple stress tests, we used the average test results with the respective pooled dispersion measure. I^2^ and Kendall’s tau were calculated as heterogeneity measures by Hunter Smith method^44,45^. The prediction intervals were calculated using the “CMA prediction intervals 1.0.0.1” software. Pooled effects were carried out using the random-effects approach, due to the inherent heterogeneity of the characteristics of the studies, such as exercises of different modalities and various stress tests. As there were not enough studies of other modalities that were not aerobic, the network analysis provided for in the protocol was not performed^26^.

The heterogeneity was explored through the search for outliers using the “externally standardized residuals” method (values farther than 1.96 standard deviations in the standardized residuals graph), the search for influential points using the difference in fits (identifying values above 1 or below −1), covariance ratio (identifying values below 1), Cook’s distance methods (identifying values far above the other studies), and subgroup analysis. Subgroup analyzes were segmented as follows: (1) exercise mode (aerobic, resistance, yoga, hand grip); (2) data presentation (peak BP defined as the maximum BP during stress, and BP variation from baseline to peak); (3) age (< 40 years, between 40 and 60 years, > 60 years); (4) sex (men, women, both); (5) population (hypertensive, normotensive, both); (6) type of stressor (include physical stressor, only mental stressor); and (7) number of stressors (unique stressor, multiple stressors).

The risk of bias assessment was carried out at the level of studies using the tool “Risk of Bias 2.0” from the Cochrane collaboration^46^ and its graphical visualization by the “R” package “robvis”^47^. Publication bias analysis was explored through a funnel plot and asymmetry hypothesis tests (Rosenthal fail-safe n, and Egger’s regression). The agreement between reviewers was estimated from Cohen’s kappa in both full-text screening (κ = 0.832; p < 0.001) and risk of bias assessment stages (κ = 0.833; p < 0.001).

## Results

### Studies characteristics

We identified 5064 studies, 5058 through structured search, and six studies through manual search, of which we included 23 full studies and one conference abstract in the analyses. The main characteristics of the studies (23 studies and one conference abstract) are shown in Table 1. Only randomized clinical trials were found, and the most frequent laboratory stress test used is the Arithmetic test (eight studies) followed by the Cold pressor test (five studies). The duration of exercise interventions varied between six and 52 weeks (an average of 18 weeks). On average, the exercise sessions had 50 min, intensities between 60–80% (moderate to high) of maximum oxygen consumption or peak heart rate, and frequency between 3 and 4 times a week. Besides that, disregarding the conference abstract, the studies included women (n = 387), men (n = 640), normotensive (n = 688), and hypertensives (n = 237; defined by either BP above 140/90 mmHg—studies prior to 2018, or BP above 130/80 mmHg—studies post 2018), in addition to 14 in whom the proportion of sexes are not clear and 116 individuals in whom the proportion of hypertensive patients are not clear.

**Table 1:** Studies characteristics.

Study	Population	Stress test	Exercise	Reactivity results
Included in qualitative and quantitative analysis				
^30^	HT, 24 women + 31 men, 48 years, sedentary, overweight, rest BP: 142/95	Public speaking Cold pressor Anger interview Mirror tracing	Aerobic (walk or cycle), 26 weeks, 03–04 times weekly, for 65 min, at 70–85% VO_2max_ (195–260 min/week)	↓SBP ↓DBP
^51^	NT, 22 men, 24 years, sedentary	Cold pressor Memory search Tone avoidance	Aerobic (run or aerobics class), 7 weeks, 4 times weekly, for 90 min, at moderate (2×) and high (2×) intensities (360 min/week)	↔ SBP ↔ DBP
^31^	60 NT + 25 HT, women, 63 years, sedentary, family caregivers, rest BP: 120/69	Public speaking	Aerobic (brisk walk), 52 weeks, 4 times weekly, for 30–40 min, at 60–75% HR_reserve_ (120–160 min/week)	↓SBP ↓DBP
^32^	NT + HT, 8 women + 17 men, 67 years, silent myocardial Ischemia, rest BP: 137/78	Anger-recall task Arithmetic Role play	Aerobic (walk), 26 weeks, 3 times weekly, for 40 min, at 70% HR_reserve_ (120 min/week)	↔ SBP ↓DBP
^33^	HT, 23 men, 41 years, rest BP: 139/92	Stroop color	Aerobic (walk or run), 12 weeks, 3 times weekly, for 45 min, at 40–50% (LI) or 70–80% (MO) VO_2max_ (135 min/week)	LI: ↓SBP e ↓DBP. MO: ↔ SBP e ↓DBP
^52^	NT, 14 women + 16 men, 22 years, sedentary, rest BP: 119/75	Arithmetic	Aerobic (run, cycle, swim, rowing, or stair climbing) or Resistance exercises, 6 weeks, 03–05 times weekly, for 40–45 min, at 70–85% HR_max_ or 8–12 repetitions (120–225 min/week)	Aerobic and resistance groups: ↓SBP e ↔ DBP
^53^	NT, 40 women + 43 men, 48 years, sedentary, rest BP: 112/65	Arithmetic	Aerobic (brisk walk or run), 26 weeks, 5 times weekly, for 47–54 min, at 65–77% HR_peak_ (235–270 min/week)	↔ SBP ↔ DBP
^54^	NT, 34 men, 25 years, sedentary, rest BP: 119/63	Stroop color	Aerobic (cycle), 12 weeks, 3 times weekly, for 30 min, at 80–90% HR_max_ (90 min/week)	↔ SBP ↔ DBP
^55^	HT, 11 women + 13 men, 64 years, rest BP: 130/73	Arithmetic Cold pressor Hand grip	Hand grip, 10 weeks, 3 times weekly, for 12 min (4 × 2’/1’), at 30% maximum voluntary isometric contraction (36 min/week)	Arithmetic and hand grip: ↓SBP ↔ DBP. Cold: ↔ SBP ↔ DBP
^56^	HT, 16 women + 14 men, 42 years, sedentary, rest BP: 144/88	Arithmetic	Aerobic (cycle), 8 weeks, 3 times weekly, for 30 min, at variable intensity (90 min/week)	↔ SBP ↔ DBP
^48^	HT, 11 women + 44 men, 43 years, rest BP: 126/82	Hand grip	Yoga, 12 weeks, 3 times weekly, for 45 min (135 min/week)	↑DBP
^49^	14 women, 36 men, 49 years	Valsalva Hand grip Tilt test	Aerobic (walk/run), 12 weeks, 3 times weekly, for 40 min, at 60–75% HR_max_ (120 min/week)	↓SBP
^37^	NT, 63 women, 56 men, 31 years, rest BP: 113/62	Arithmetic Stroop color Orthostatic	Aerobic (12 weeks,4 times weekly, for 40-55 min, at 55–80% HR_max_)	↔ SBP ↔ DBP
^50^	HT, 14 patients, rest BP: 153/102	Hand grip Tilt test	Yoga (6 weeks, 6 times weekly, for 30 min)	Hand grip: ↓SBP ↔ DBP, Tilt test: ↓SBP ↓DBP
^57^	NT, 60 men, 18–35 years, rest BP: 125/72	Hand grip	Aerobic (walk/run, 12 weeks, alternate days, for 30 min, at 55–69% HR_max_)	Hand grip: ↔ SBP ↓DBP
Included only in qualitative analysis				
^29^	NT, 37 men, 42 years, rest BP: 123/75	Arithmetic	12 weeks of aerobic (walk or run, 3 times weekly, for 50 min, at 70% VO_2max_) or resistance (2 times weekly, 20 min of flexibility + 30 min of resistance exercise circuit) (150 min/week)	↓SBP ↓DBP
^58^	NT, 46 pre- and post-menopausal women, 50 years, rest BP: 114/69	Public speaking Cold pressor	12 weeks of aerobic (walk or run, 3 times weekly, for 50 min, at 70% VO_2max_) or resistance (2 times weekly, 20 min of flexibility + 30 min of resistance exercise circuit) (150 min/week)	Cold: postmenopausal ↔ SBP ↓DBP, premenopausal ↔ SBP ↔ DBP. speech: both ↔ SBP ↔ DBP
^34^	16 NT + 11 HT, men, 41 years, rest BP: 135/89	Attention task	12 weeks of aerobic (walk, run, or stair climbing, 3 times weekly, for 50 min, at 70% VO_2max_) or resistance (2 times weekly, 20 min of flexibility + 30 min of resistance exercise circuit) (150 min/week)	↔ SBP ↓DBP
^35^	NT, 36 men, 44 years, rest BP: 129/85	Arithmetic	12 weeks of aerobic (walk, run or stair climbing, 3 times weekly, for 50 min, at 70% VO_2max_) or resistance (2 times weekly, 20 min of flexibility + 30 min of resistance exercise circuit) (150 min/week)	Aerobic: ↓SBP ↓DBP Control: ↔ SBP ↓DBP
^36^	NT, 24 men, 33 years, sedentary, rest BP: 124/73	Cold pressor Memory search Tone avoidance	Aerobic (run, jump, stair climbing, soccer or basketball), 32 weeks, self-selected frequency, for 120 min, at 70% HR_max_ (at least 120 min/week)	↔ SBP ↔ DBP
^59^	NT, 38 women + 50 men, rest BP: 129/76	Trier social stress test	Aerobic, 3 times weekly, 26 weeks, 45–60 min, 75% HR_peak_ (135–180 min/week)	↔ SBP ↔ DBP
^60^	3 women + 38 men, Firefighters, rest MBP: 96	Video-based strategy and tactics drill	Aerobic (rowing), 16 weeks, 4 times weekly, for 40 min, at variable intensity (160 min/week)	↓MBP
^61^	NT, 14 women + 19 men, 51 years, obstructive sleep apnea, rest BP: 122/79	Stroop color	Aerobic (cycle), 24 weeks, 3 times weekly, 5 min of stretching + 40 min of cycling (anaerobic threshold up to the respiratory compensation point) + 10 min of strengthening + 5 min of cool down	↔ MBP
^62^^a^	NT, 80 participants, 18–50 years, healthy	Cold pressor	Yoga or aerobic (swim), 12 weeks	Cold: ↓SBP ↔ DBP

The age refers to the average.

*BP* blood pressure, *SBP* systolic blood pressure, *DBP* diastolic blood pressure, *MBP* mean blood pressure, *HR* heart rate, *HT* hypertensives, *NT* normotensives, *LI* low intensity, *MO* moderate intensity.

^a^Only conference abstract available.

Regarding exercise mode, 21 studies referred to aerobic training, 2 to yoga training, 5 to resistance training, and 1 to isometric handgrip training. Considering the main results of the 23 studies, 9 found significative reductions in systolic (SBP), 10 in diastolic (DBP), and 1 in mean BP reactivity. On the other hand, only 1 study found a worsening of DBP reactivity with Yoga intervention^48^. As for the BP measurement method, two studies used the auscultatory approach^33,34^, one used continuous BP measurement on the finger^37^, two did not specify the type of BP measurement used^49,50^, and all the others used the oscillometric method^29–32,35,36,48,51–61^. We did not identify exercise characteristics that distinguish studies with significant and non-significant responses based on qualitative analyses. However, as for the population, most of the studies with favorable results include hypertensive patients.

### Meta-analysis results

Some characteristics motivated the inclusion of certain studies only in the qualitative analyses, among which we highlight: (1) four of the studies (published from 1988 to 1991) with aerobic exercises^29,34,35,58^ had as a comparator group individuals that trained flexibility and resistance circuit exercises with volume, frequency, and intensities smaller than the aerobic. In addition, a study compared swimming and yoga training^62^. So, they used an active comparator with exercise and therefore were disregarded in the meta-analysis. (2) Some studies^34–36,59^ did not present dispersion measures and were also not included in the quantitative analysis. (3) In addition to these, we found an abstract presented at scientific event, of which we have not identified the related full-text publication^62^, it reported decreased reactivity of SBP but not DBP to the cold pressor test after 12 weeks of yoga compared to swimming. This conference abstract was not included in the meta-analytic analysis. (4) Finally, two studies reported results only in mean BP, therefore, they were not included in the meta-analysis^60,61^.

**Figure 2:**
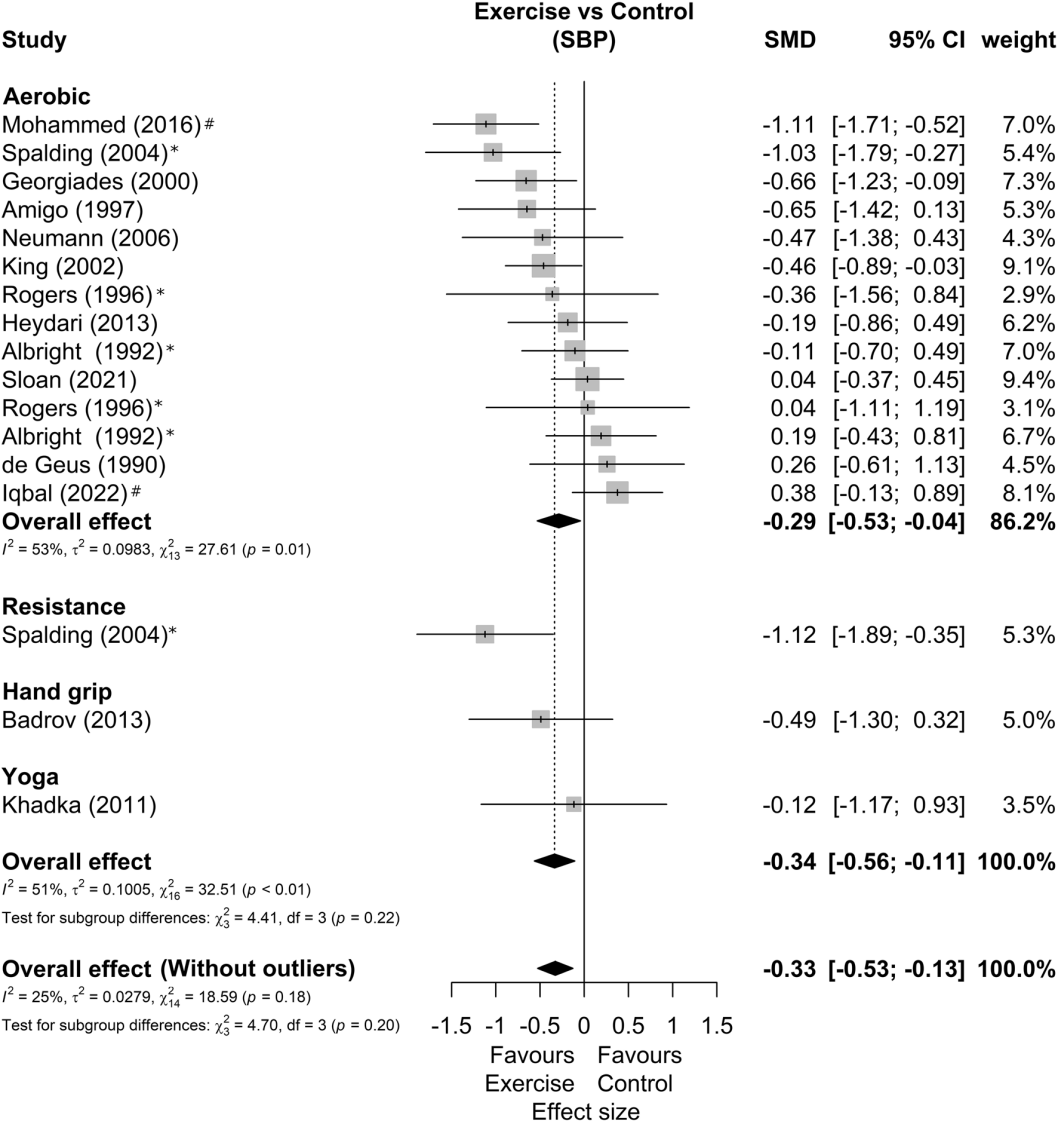
Systolic blood pressure reactivity forest plot segmented by exercise mode. Asterisk: studies with more than one comparison; hash: possible outlier or influential point; *SMD* standardized mean difference, *SBP* systolic blood pressure, *CI* confidence interval.

**Figure 3:**
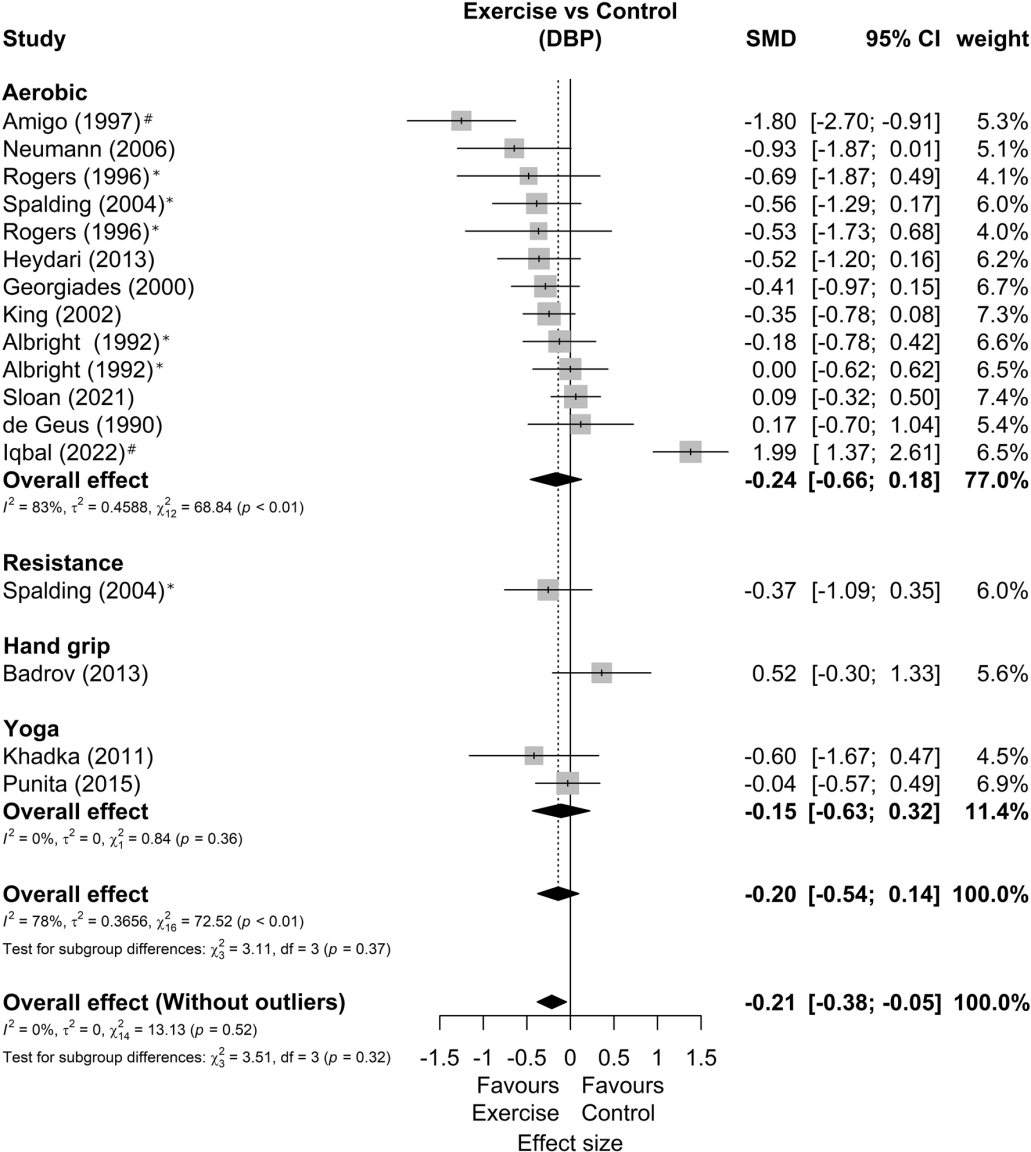
Diastolic blood pressure reactivity forest plot segmented by exercise mode. Asterisk: studies with more than one comparison; hash: possible outlier or influential point; SMD standardized mean difference, *DBP* diastolic blood pressure, *CI* confidence interval.

Among 15 studies included in the meta-analysis, three presented two possible comparisons with a control group without exercise according to exercise intensity^33^, exercise mode^52^, and sex^53^. In addition, one study only shows results for SBP^49^ and another only for DBP^48^, resulting in 14 studies and 17 comparisons analyzed in each outcome. Regarding the characteristics of the exercise, the duration, frequency, volume, and intensities agree with the qualitative phase. The forest plots of SBP and DBP reactivity segmented by exercise mode are present in Figs. 2 and 3*,* respectively. We found a small favorable effect of exercise in SBP response (SMD = −0.34 [−0.56; −0.11]; Prediction Intervals = −1.06 to 0.38; representing average reductions of 2.5 ± 3.6 mmHg) and null effect on DBP responses (SMD = −0.20 [−0.54; 0.14]; Prediction Intervals = −1.54 to 1.14, representing average reductions of 2.0 ± 3.5 mmHg). Sensitivity analyses showed that two studies^56,57^ could be outliers and influential points. The analysis omitting these studies showed SBP effect of −0.33[−0.53; −0.13] and DBP effect of −0.21 [−0.38; −0.05].

**Figure 4:**
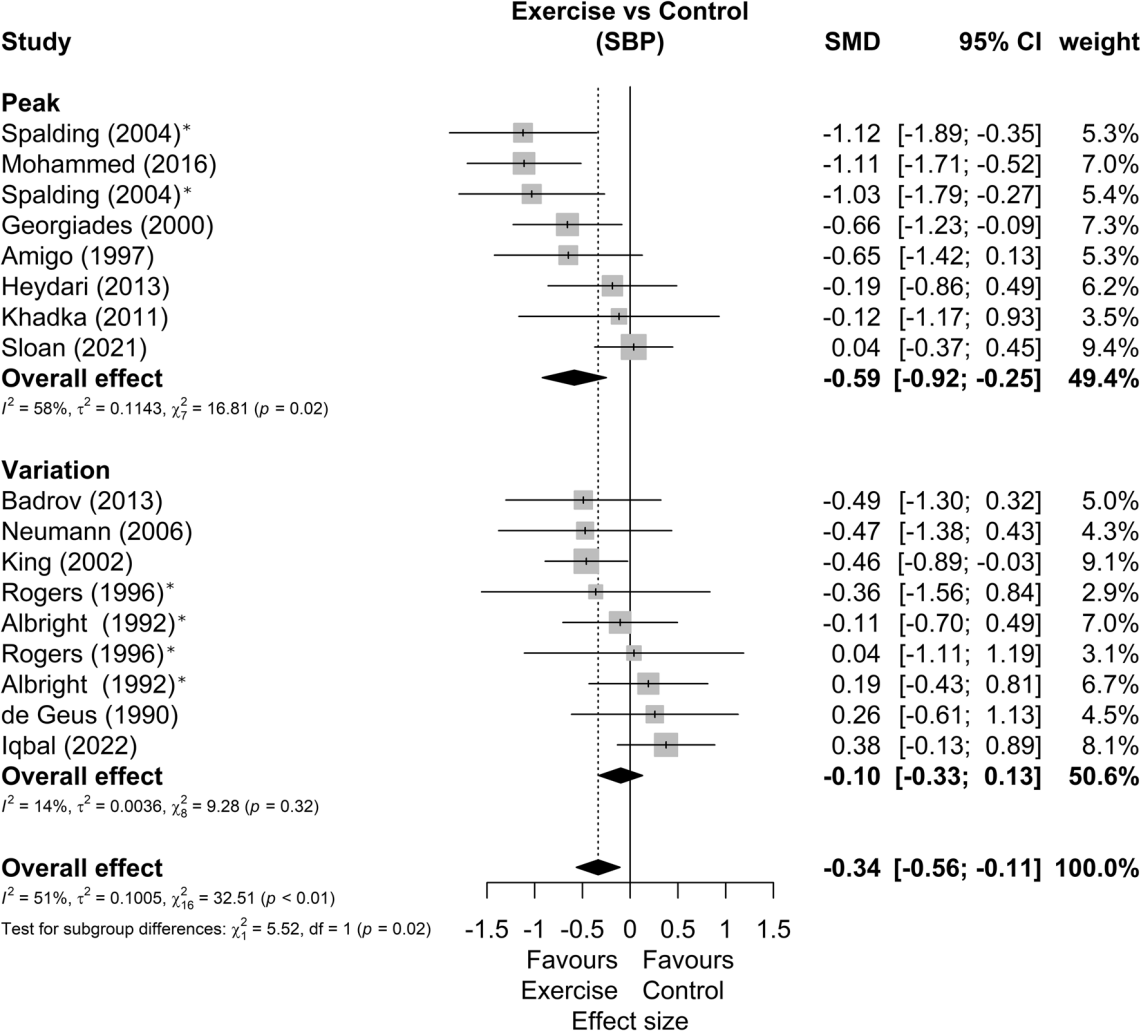
Systolic blood pressure reactivity forest plot segmented by data presentation (peak BP defined as the maximum BP during stress, or BP variation from baseline to peak). Asterisk: studies with more than one comparison; hash: possible outlier or influential point; *SMD* standardized mean difference, *SBP* systolic blood pressure, *CI* confidence interval.

**Figure 5:**
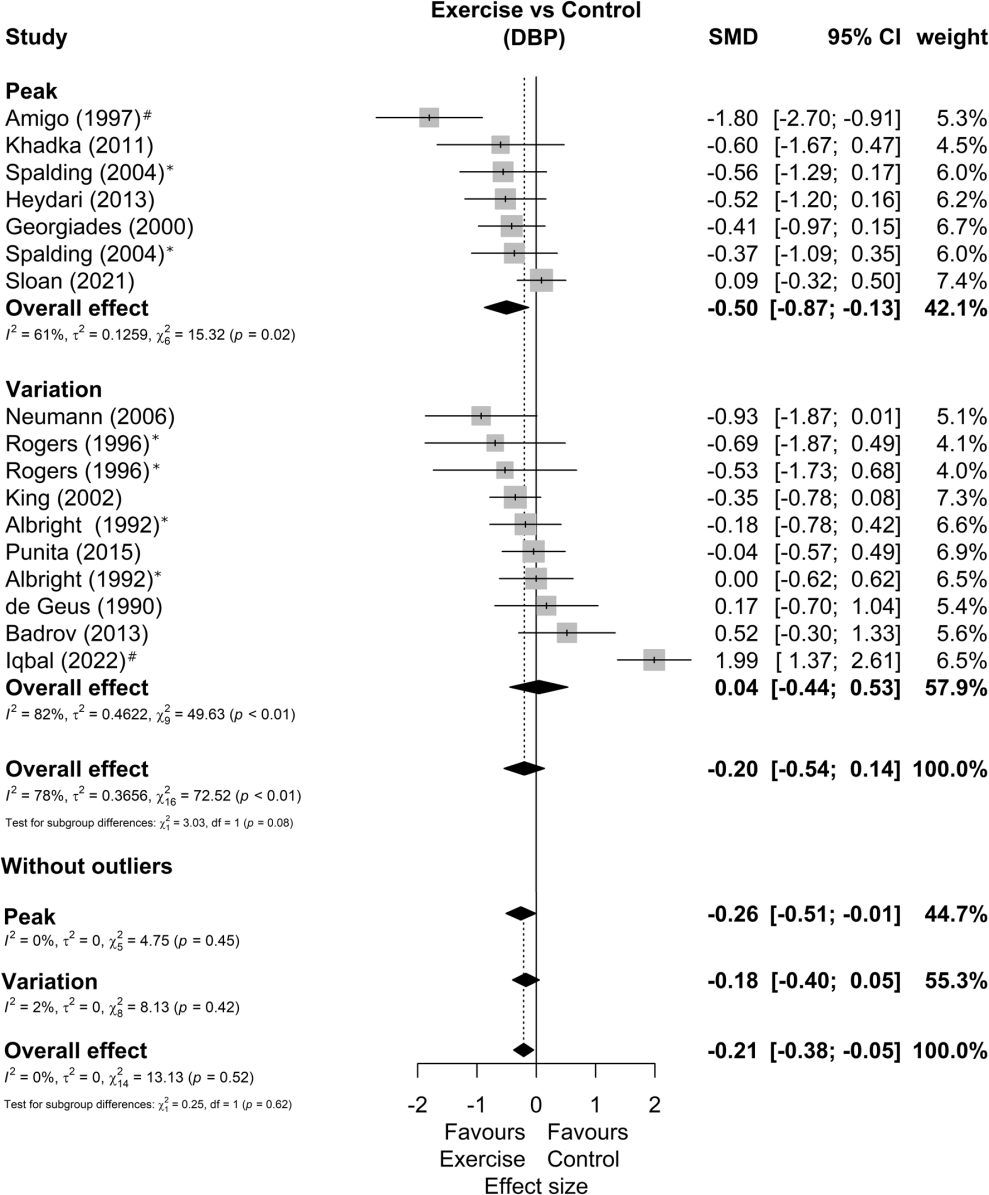
Diastolic blood pressure reactivity forest plot segmented by data presentation (peak BP defined as the maximum BP during stress, or BP variation from baseline to peak). Asterisk: studies with more than one comparison; hash: possible outlier or influential point; *SMD* standardized mean difference, *DBP* diastolic blood pressure, *CI* confidence interval.

The forest plots of SBP and DBP reactivity segmented by data presentation (peak BP or BP variation from baseline) are present in Figs. 4 and 5*,* respectively. In this sense, we found subgroup differences (p = 0.02) between BP reactivity data presentation methods in the SBP, with studies that reported peak stress having moderate effects favorable to exercise (SMD = −0.59 [−0.92; −0.25]; Prediction Intervals = −1.52 to 0.34), while studies reporting baseline variation have null effects (SMD = −0.10 [−0.33; 0.13]; Prediction Intervals = −0.41 to 0.21). As for DBP, we found no differences between the subgroups (p = 0.08). Although the subgroup that presented the stress peak had a moderate effect (SMD = −0.50 [−0.87; −0.13]) and the subgroup that presented the data as baseline variation had null effects (SMD = 0.04 [−0.44; 0.53]). Sensitivity analyses showed that two studies^56,57^ could be outliers and influential points. The analysis omitting these studies showed similar effects between studies that presented the data as peak stress (SMD = −0.26 [−0.51; –0.01]) or as variation from baseline (SMD = −0.18 [−0.40; −0.05]).

Other sensitivity/subgroup analyses were performed considering only studies with aerobic training since these represented a large part of the sample. When comparing the studies by age we find no significant differences in SBP (p = 0.26) or DBP (p = 0.22) reactivity. However, when comparing the studies by sex, women present larger effects than men in SBP (p < 0.01), with no differences in DBP (p = 0.59). When comparing studies by population (only normotensive, only hypertensive, or both) we found significant differences with greater effects in hypertensive patients in the reactivity of SBP (p < 0.01) and DBP (p = 0.05). Lastly, when comparing the studies by type or the number of stressors, we did not find significant differences in the SBP reactivity (p = 0.69 and p = 0.47 respectively). However, in DBP there was a significant difference between the types of stressors, with greater effects in studies with only mental stressors (p = 0.03), but without significant effects of the number of stressors (p = 0.82).

In general, prediction intervals show great heterogeneity, crossing the null effect. We observed greater heterogeneity in DBP analyses, that included the three biggest prediction intervals: physical stressor (from −3.44 to 4.34), men (from −3.66 to 3.94), and participants younger than 40 years (from −2.63 to 3.13). The greater heterogeneity in the DBP subgroups in comparison with SBP is further reinforced by the other heterogeneity measures, such as I^2^ and τ^2^. The summary of these analyzes can be seen in Table 2.

**Table 2:** Subgroup analysis for blood pressure reactivity.

Subgroup variables	Effect size				Subgroup differences p	Heterogeneity			
	SMD [95% IC]	Weight (%)	k	m		I^2^ (%)	τ^2^	Q	Prediction intervals
SBP age									
< 40 years	−0.06 [−0.43; 0.31]	39.0	5	5	0.26	59	0.083	9.78*	−1.16; 1.04
Between 40 and 60 years	−0.42 [−0.77; −0.07]	45.5	5	7		48	0.082	11.55	−1.29; 0.45
> 60 years	−0.46 [−0.85; −0.07]	15.6	2	2		0	0.000	0.00	–
SBP sex									
Men	0.13 [−0.21; 0.47]	28.6	4	5	< 0.01	0	0.000	2.5	–
Women	−0.72 [−1.15; −0.28]	18.7	2	2		67	0.032	3.02	–
Both	−0.31 [−0.61; −0.01]	52.7	6	7		47	0.058	11.27	−1.04; 0.42
SBP population									
Hypertensive only	−0.54 [−0.94; −0.13]	21.4	3	4	< 0.01	0	0.000	1.3	–
Normotensive only	−0.02 [−0.29; 0.25]	54.9	6	7		42	0.042	10.27	−0.65; 0.61
Both	−0.66 [−1.00; −0.32]	23.7	3	3		38	0.006	3.2	−3.07; 1.75
SBP stressor type									
Include physical stressor	−0.23 [−0.71; 0.25]	42.2	5	5	0.69	79	0.209	18.71*	−1.93; 1.41
Only mental stressor	−0.33 [−0.56; −0.10]	57.8	7	9		2	0.000	8.14	–
SBP number of stressors									
Unique stressor	−0.21 [−0.49; 0.07]	62.2	5	5	0.47	43	0.60	14.0	−2.70; 2.28
Multiple stressors	−0.40 [−0.84; 0.03]	37.8	7	9		69	0.140	12.76*	−1.43; 0.63
Overall SBP	−0.29 [−0.53; −0.04]	100	12	14		53	0.098	27.61*	−1.03; 0.45
DBP age									
< 40 years	0.25 [−0.53; 1.02]	40.5	5	5	0.22	90	0.664	40.5*	−2.63; 3.13
Between 40 and 60 years	−0.52 [−0.95; −0.08]	43.5	4	6		57	0.134	11.76*	−1.71; 0.67
> 60 years	−0.45 [−0.84; −0.06]	15.9	2	2		16	0.000	1.19	–
DBP sex									
Men	0.14 [−0.89; 1.17]	34.7	4	5	0.59	89	1.149	37.98*	−3.66; 3.94
Women	−0.35 [−0.78; 0.08]	9.2	1	1		0	0.000	0.00	–
Both	−0.43 [−0.81; −0.05]	56.1	6	7		66	0.148	17.71*	−1.54; 0.68
DBP population									
Hypertensive only	−0.83 [−1.40; −0.26]	26.8	3	4	0.05	56	0.127	6.84	−2.81; 1.15
Normotensive only	0.15 [−0.42; 0.72]	57.3	6	7		86	0.481	42.66*	−1.78; 2.08
Both	−0.45 [−0.84; −0.06]	15.9	2	2		16	0.000	1.19	–
DBP stressor type									
Include physical stressor	0.45 [−0.38; 1.29]	33.3	4	4	0.03	92	0.632	35.038*	−3.44; 4.34
Only mental stressor	−0.51 [−0.80; −0.22]	66.7	7	9		39	0.056	13.04	−1.17; 0.15
DBP number of stressors									
Unique stressor	−0.25 [−0.86; 0.36]	68.3	7	9	0.82	87	0.707	63.52*	−2.37; 1.87
Multiple stressors	−0.17 [−0.52; 0.18]	31.7	4	4		44	0.029	5.31	−1.23; 0.89
Overall DBP	−0.24 [−0.66; 0.18]	100	11	13		83	0.459	68.84*	−1.80; 1.32

Include physical stressor: studies that used only physical stressors or in conjunction with mental stressors.

*SBP* systolic blood pressure, *DBP* diastolic blood pressure, *SMD* effect size by standardized mean differences, *CI* confidence interval, *k* number of studies, *m* number of comparisons, *I*^2^ Higgins e Thompson *I*^2^, *Q* Cochran’s *Q*, *τ*^2^ Kendall’s *τ*^2^.

*p < 0.05.

### Quality and bias assessment

**Figure 6:**
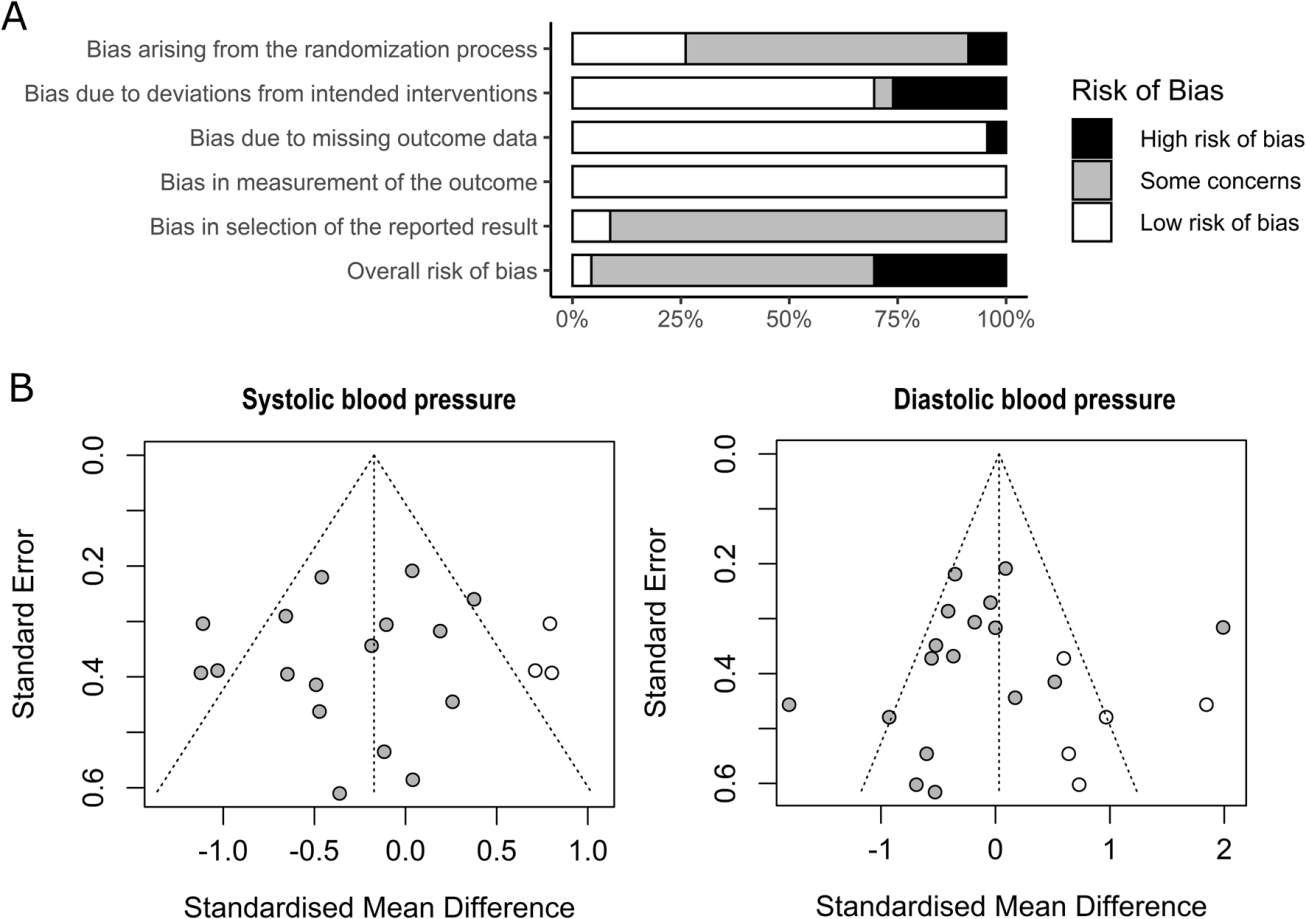
(**A**) Risk of bias summary (k = 23). (**B**) Publication risk of bias funnel plots.

The graphical summary of the risk of bias assessment is shown in Fig. 6A. This analysis excludes the study that was only been presented at a scientific event without publishing the full-text^63^. Although seven studies were classified as having a high risk of bias, only two of them^30,32^ were included in the quantitative analysis. Furthermore, the omission of these studies did not significantly change the results (SBP SMD = −0.30 [−0.55; −0.05]; and DBP SMD = −0.15 [−0.52; 0.23]). When considering only studies included in the meta-analyses, we reduced the high risk of bias from 7 of 23 to 2 of 15 studies (30% to 13% of the total).

It is worth mentioning that none of the studies were participants blinded to interventions, as this is difficult to do with exercise interventions. In addition, just three studies mention previous protocols, clinical study records, or analysis plans, which could prevent the self-selection of analyzes and results (i.e., reporting bias). One of the studies presented an intention-to-treat analysis^37^ and no authors reported any conflicts of interest.

The publication bias tests showed no asymmetries in the funnel plot for SBP (Egger regression p = 0.475; Fail safe n = 101) or DBP reactivity (Egger regression p = 0.331; Fail-safe n = 62). However, three omitted results are expected by trim and fill funnel plots in SBP and five in DBP (Fig. 6B).

## Discussion

Our results showed that most of the studies (64%) showed favorable BP responses (either in SBP or DPB) after exercise training, and the most frequent stressor test was the arithmetic task (eight studies) which might not reflect daily activities. The quantitative analysis suggests a small effect of exercise training attenuating SBP and DBP reactivity to stress (SBD effect size = −0.33 [−0.53; −0.13] and DBP effect size = −0.21 [−0.38; −0.05] without outliers). However, the available data about non-aerobic activities are quite limited and, therefore, do not allow us to interpret properly nor explore subgroup effects.

Concerning the risk of bias in the included studies, biases related to deviations from the intended interventions account for most of the high risk of bias ratings. In general, the reason for the high risk of bias in these studies is the same reason that motivated their non-inclusion in the quantitative analysis, especially the inadequate control groups. Specifically, they had comparator groups that trained in resistance circuit exercises with volume, frequency, and intensities lower than the aerobic^29,34,35,58^. Originally, these comparators were treated as controls, as it was considered that they would not significantly influence the cardiovascular system^34^. Nevertheless, there is evidence that resistance exercise influences the cardiovascular function^64^, so we excluded these studies from the quantitative analysis.

We also emphasize that no study has blinded participants regarding the interventions. However, we believe that this feature did not result in a higher risk of bias in exercise training trials^65,66^. Besides that, although they were described as randomized, they do not describe this process with a sufficient level of detail and do not present records of protocols, analysis plans, or clinical study records, so the evaluation of the selection of reported results bias was compromised. In summary, the studies included were generally of satisfactory quality but with some concerns that should be polished in later studies^67^.

As for the different forms of data presentation, the studies that reported peak stress in SBP showed moderate effects favorable to exercise (SMD = −0.59 [−0.92; −0.25]), while studies reporting variation in the baseline showed null effects (SMD = −0.10 [−0.33; 0.13]). This may be an indication that physical exercise, more than reducing the stress response, lowers resting BP and given the same magnitude of stress response, lowers peak BP. Despite this, we highlight the clinical importance of reducing BP peaks, especially given their relationship with the risk of stroke^68,69^.

In general, SMD pooled effects showed high heterogeneity, variating from −0.83 to 0.45, depending on the sub-analysis. Also, prediction intervals show great heterogeneity, especially in DBP. Interestingly, all higher heterogeneities are resolved by omitting a single study^57^. This omission decreases the prediction intervals of the higher heterogenic subgroups: DBP of patients younger than 40 years (prediction intervals from [−2.63; 3.13] to [−0.85; 0.59], and I^2^ from 90 to 27%), DBP of men (prediction intervals from [−3.66; 3.94] to [−0.82; 0.10], and I^2^ from 89 to 0%), and DBP in studies that include physical stressors (prediction intervals from [−3.44; 4.34] to [0.36; 0.26], and I^2^ from 92 to 13%). Although we did not identify a characteristic of this study that differentiates from the others, this study was the only one to present DBP change values during a stressor favorable to the control group (4 mmHg below the exercise group).

As for SBP, the greatest heterogeneities were found in the prediction intervals of: (1) subgroup with both hypertensive and normotensive individuals, which was expected given the non-homogeneity of this subgroup; and (2) subgroup with a single stressor, which despite not including multiple stressors, can be explained by the wide variety of stressor tests included, that can involve physiological, environmental, emotional, and cognitive stressors, and may involve social-evaluative threat, uncontrollability, and unpredictability^3^.

These different methods can act by different mechanisms, which may explain even the subgroups difference between studies that included physical stressors or only mental stressors. Interestingly they seem to impact only DBP (p = 0.03) but not SBP (p = 0.69). As an example of the different mechanisms of action to increase BP, a physical stressor (i.e. cold pressor test) seems to act through arteriolar vasoconstriction^70^, by sympathetic adreno-medullary axis activation but minimal hypothalamic–pituitary–adrenal axis stimulation^3^. A mental stressor (i.e. Stroop color and word test) could cause an increase in heart rate and pulse pressure with no changes in the stroke volume, in vascular resistance nor did it affect central arterial wave reflection^71^, showing less vascular protagonism. Besides that, literature has shown that affective state^11^, self-efficacy^72^, familiarity with the tests, and the application moment of the tests after the exercise training could be confounding factors that would explain also part of the heterogeneity^22^. These pieces of information are not well described in most exercise training studies, which did not allow us to use them in the sensitivity analyses of the present study.

As for the subgroup analyses, our results bring information in line with and sometimes disparate from the current literature, especially regarding the characteristics of the population. Contrary to the literature, our results did not find differences when age classes were compared (SBP p = 0.26; DBP p = 0.22). However, the younger subgroup showed null effects while the other groups showed favorable results for exercise. Furthermore, when we analyze the prediction intervals, only the older group presents a favorable result for exercise interventions. Further, although the literature shows that men have a more exacerbated reactivity than women^73,74^, our subgroup analysis shows favorable results on the SBP of women compared to men (p < 0.01), and similar responses between sexes on the DBP (p = 0.59). However, the favorable result for women in SBP may be inconsistent, given that there are only two studies in this subgroup, one of them being the best result found among all SBP studies^49^.

Lastly, hypertensive individuals seem to have greater vascular and lower cardiac output responses^71^, and antihypertensive drugs could alter stress reactivity responses, although this is still a little explored aspect^75^. In this sense, the greatest effect in hypertensive patients was confirmed in the present study subgroup analysis, either in SBP (p < 0.01) or DBP (p = 0.05).

One significant implication of our study is the identification of the gap in the literature on the effect of different exercise training modalities on peak BP responses. One might understand that breathing exercises or yoga might represent a low risk for peak BP responses, however, resistance training, CrossFit, and high-intensity interval training are some of the modalities that are in high demand and worth exploring. Although the literature still does not show what is the minimal clinically important difference in response to stress, studies have shown that BP reactivity is associated with the development of hypertension^4,5,7^, future cardiovascular events^2,3^, and it is a good predictor of left ventricular mass^6^, therefore being a clinical marker independent of resting BP. Therefore, the attenuation of BP reactivity through simple stressor tasks may indicate a reduction in cardiovascular risk in the clinical routine. Besides, based on the present systematic review findings, aerobic exercises are demonstrated to be potential strategies capable of reducing BP reactivity (mainly in SBP), with similar magnitude to the previous meta-analysis about the effects of one session of exercise (SBP effect size = −0.38 [−0.49; −0.27]; DBP effect size = −0.51 [−0.70; −0.33])^23^. This added to the fact that exercise training reduces several risk factors^76^, including the ability to reduce resting BP in hypertensive patients^77^, making physical exercise the protagonist of clinical interventions focusing on BP responses and the reduction of cardiovascular risk.

It is worth noting that the present study has some limitations, such as the high statistical heterogeneity found for most of our analyses, which could lead to doubtful internal validity. Second, we experienced a lack of statistical power due to the low number of interventional arms (k = 1 for some subgroups) which limits interpretation. In addition, there are limitations from the included studies, such as the wide variety of stress tests used, which makes it challenging to understand the patterns of response to each type of stress and might increase the heterogeneity across studies. Besides that, most of the included studies performed aerobic exercises, limiting the understanding of the results for other types of exercise.

As future directions, we encourage exploring the effects of non-aerobic exercise modalities, especially traditional resistance training and Pilates^78^, in addition to studies involving stressors with more remarkable similarity to daily situations, involving different sensations (e.g., pain, cold, heat, tiredness, loss of control, pressure for performance, frustration, fear, anger). In this sense, Augmented Reality or Virtual Reality can be used as strategies to generate stress close to daily life in a safe and controlled way. Also, even prolonged stressors such as those found at work or in competitive sporting environments could be explored. A good example of a study that proposed a stress test appropriated to the reality of its studied population was from Throne et al.^60^, which was carried out with firefighters, and used a video test that presents risk situations in which they should make difficult decisions in a limited time.

## Conclusions

In summary, there is evidence that aerobic exercise training helps mitigates systolic blood pressure reactivity to laboratory-based stress tests, especially in hypertensive subjects. However, considering the small effect size, and the large confidence intervals of the pooled effect, the clinical relevance for some subgroups must be taken with caution. Future studies should consider exploring the different aspects of the population’s characteristics, type of stress testing, and other exercise modalities, especially resistance training. Lastly, we highlight the importance of data presentation in two main ways: peak BP and BP variation from baseline, as these approaches allow complementary analyses.

## Data Availability

The datasets generated during and/or analysed during the current study are available from the corresponding author on reasonable request.

## References

[1] Chrousos G (2009). Stress and disorders of the stress system. Nat. Rev. Endocrinol..

[2] Turner AI (2020). Psychological stress reactivity and future health and disease outcomes: A systematic review of prospective evidence. Psychoneuroendocrinology.

[3] Bali, A. & Jaggi, A. S. Clinical experimental stress studies: methods and assessment. *Rev. Neurosci.* **26,** 555 (2015). 10.1515/revneuro-2015-0004 26020552

[4] Matthews KA, Woodall KL, Allen MT (1993). Cardiovascular reactivity to stress predicts future blood pressure status. Hypertension.

[5] Matthews KA, Salomon K, Brady SS, Allen MT (2003). Cardiovascular reactivity to stress predicts future blood pressure in adolescence. Psychosom. Med..

[6] Georgiades A, Lemne C, de Faire U, Lindvall K, Fredrikson M (1996). Stress-induced laboratory blood pressure in relation to ambulatory blood pressure and left ventricular mass among borderline hypertensive and normotensive individuals. Hypertension.

[7] Wood DL, Sheps SG, Elveback LR, Schirger A (1984). Cold pressor test as a predictor of hypertension. Hypertension.

[8] Myers B (2017). Corticolimbic regulation of cardiovascular responses to stress. Physiol. Behav..

[9] Gianaros PJ, Wager TD (2015). Brain–body pathways linking psychological stress and physical health. Curr. Dir. Psychol. Sci..

[10] Gerra G (2001). Neuroendocrine responses to experimentally-induced psychological stress in healthy humans. Psychoneuroendocrinology.

[11] Brummett BH, Boyle SH, Kuhn CM, Siegler IC, Williams RB (2009). Positive affect is associated with cardiovascular reactivity, norepinephrine level, and morning rise in salivary cortisol. Psychophysiology.

[12] Jung YP (2017). Effects of ingesting a pre-workout dietary supplement with and without synephrine for 8 weeks on training adaptations in resistance-trained males. J. Int. Soc. Sports Nutr..

[13] Foley P, Kirschbaum C (2010). Human hypothalamus–pituitary–adrenal axis responses to acute psychosocial stress in laboratory settings. Neurosci. Biobehav. Rev..

[14] Herman, J. P. *et al.**Comprenensive Physiology*. 603–621 (Wiley, 2016). 10.1002/cphy.c150015

[15] Hermans EJ, Henckens MJAG, Joëls M, Fernández G (2014). Dynamic adaptation of large-scale brain networks in response to acute stressors. Trends Neurosci..

[16] van Oort J (2017). How the brain connects in response to acute stress: A review at the human brain systems level. Neurosci. Biobehav. Rev..

[17] Smeets T (2010). Autonomic and hypothalamic–pituitary–adrenal stress resilience: Impact of cardiac vagal tone. Biol. Psychol..

[18] Castaldo R (2015). Acute mental stress assessment via short term HRV analysis in healthy adults: A systematic review with meta-analysis. Biomed. Signal Process. Control.

[19] Walker FR, Pfingst K, Carnevali L, Sgoifo A, Nalivaiko E (2017). In the search for integrative biomarker of resilience to psychological stress. Neurosci. Biobehav. Rev..

[20] Appelhans BM, Luecken LJ (2006). Heart rate variability as an index of regulated emotional responding. Rev. Gen. Psychol..

[21] Whelton PK (2018). Clinical Practice Guideline 2017 ACC/AHA/AAPA/ABC/ACPM/AGS/APhA/ASH/ASPC/NMA/PCNA guideline for the prevention, detection, evaluation, and management of high blood pressure in adults a report of the American College of Cardiology. Hypertension.

[22] Hamer M, Taylor A, Steptoe A (2006). The effect of acute aerobic exercise on stress related blood pressure responses: A systematic review and meta-analysis. Biol. Psychol..

[23] Mariano IM, Amaral AL, Ribeiro PAB, Puga GM (2022). A single session of exercise reduces blood pressure reactivity to stress: A systematic review and meta-analysis. Sci. Rep..

[24] Huang C-J, Webb HE, Zourdos MC, Acevedo EO (2013). Cardiovascular reactivity, stress, and physical activity. Front. Physiol..

[25] Jackson EM, Dishman RK (2006). Cardiorespiratory fitness and laboratory stress: A meta-regression analysis. Psychophysiology.

[26] Mariano IM, Amaral AL, Puga GM (2020). Protocol of a systematic review with network meta-analysis: Chronic effects of physical exercise on blood pressure responsiveness to non-cardiopulmonary stress tests. Protocols.

[27] Moher D, Liberati A, Tetzlaff J, Altman DG (2009). Preferred reporting items for systematic reviews and meta-analyses: The PRISMA statement. PLoS Med..

[28] Page, M. *et al.* The PRISMA 2020 statement: An updated guideline for reporting systematic reviews. *BMJ* **29,** 1–36 (2020). 10.31222/osf.io/v7gm2. 10.1186/s13643-021-01626-4 PMC8008539 33781348

[29] Blumenthal JA (1990). Aerobic exercise reduces levels of cardiovascular and sympathoadrenal responses to mental stress in subjects without prior evidence of myocardial ischemia. Am. J. Cardiol..

[30] Georgiades A (2000). Effects of exercise and weight loss on mental stress-induced cardiovascular responses in individuals with high blood pressure. Hypertension.

[31] King, A. C., Baumann, K., O’Sullivan, P., Wilcox, S. & Castro, C. Effects of moderate-intensity exercise on physiological, behavioral, and emotional responses to family caregiving: A randomized controlled trial. *J. Gerontol.Ser. A Biol. Sci. Med. Sci.* **57**, 26–36 (2002). 10.1093/gerona/57.1.m26 11773209

[32] Neumann SA, Brown JRP, Waldstein SR, Katzel LI (2006). A walking program’s attenuation of cardiovascular reactivity in older adults with silent myocardial ischemia. J. Aging Phys. Act..

[33] Rogers MW, Probst MM, Gruber JJ, Berger R, Boone JBJ (1996). Differential effects of exercise training intensity on blood pressure and cardiovascular responses to stress in borderline hypertensive humans. J. Hypertens..

[34] Sherwood A, Light KC, Blumenthal JA (1989). Effects of aerobic exercise training on hemodynamic responses during psychosocial stress in normotensive and borderline hypertensive type A men: A preliminary report. Psychosom. Med..

[35] Blumenthal JA (1988). Exercise training in healthy type A middle-aged men: Effects on behavioral and cardiovascular responses. Psychosom. Med..

[36] de Geus EJ, van Doornen LJ, Orlebeke JF (1993). Regular exercise and aerobic fitness in relation to psychological make-up and physiological stress reactivity. Psychosom. Med..

[37] Sloan RP (2021). The impact of aerobic training on cardiovascular reactivity to and recovery from psychological and orthostatic challenge. Psychosom. Med..

[38] Balduzzi S, Rücker G, Schwarzer G (2019). How to perform a meta-analysis with R: A practical tutorial. Evid. Based Ment. Heal..

[39] Viechtbauer W (2010). Conducting meta-analyses in R with the metafor package. J. Stat. Softw..

[40] Takeshima N (2014). Which is more generalizable, powerful and interpretable in meta-analyses, mean difference or standardized mean difference?. BMC Med. Res. Methodol..

[41] Andrade, C. Mean difference, standardized mean difference (SMD), and their use in meta-analysis. *J. Clin. Psychiatry* **81**, 13681 (2020). 10.4088/JCP.20f13681 32965803

[42] Luo D, Wan X, Liu J, Tong T (2018). Optimally estimating the sample mean from the sample size, median, mid-range, and/or mid-quartile range. Stat. Methods Med. Res..

[43] Wan X, Wang W, Liu J, Tong T (2014). Estimating the sample mean and standard deviation from the sample size, median, range and/or interquartile range. BMC Med. Res. Methodol..

[44] Schmidt, F. L. & Hunter, J. E. *Methods of Meta-analysis: Correcting Error and Bias in Research Findings*. (SAGE Publications, 2015). 10.4135/9781483398105.

[45] Petropoulou M, Mavridis D (2017). A comparison of 20 heterogeneity variance estimators in statistical synthesis of results from studies: A simulation study. Stat. Med..

[46] Higgins, J. P., Savović, J., Page, M. J. & Sterne, J. A. RoB 2: A revised Cochrane risk-of-bias tool for randomized trials. *BMJ. *1–24 (2019). https://methods.cochrane.org/ (**in press**).

[47] McGuinness, L. A. & Higgins, J. P. T. Risk‐of‐bias VISualization (robvis): An R package and Shiny web app for visualizing risk‐of‐bias assessments. *Res. Synth. Methods* (2020). 10.1002/jrsm.1411. 10.1002/jrsm.1411 32336025

[48] Punitha P (2016). Randomized controlled trial of 12-week yoga therapy as lifestyle intervention in patients of essential hypertension and cardiac autonomic function tests. Natl. J. Physiol. Pharm. Pharmacol..

[49] Mohammed MA, Rahmy AF, Mohamed GS, Kaddah AF (2016). Effect of exercise training on cardiovascular responses in diabetic autonomic neuropathy. Int. J. PharmTech Res..

[50] Khadka R, Paudel BH, Sharma VP, Kumar S, Bhattacharya N (2010). Effect of yoga on cardiovascular autonomic reactivity in essential hypertensive patients. Health (San Fr.).

[51] de Geus EJ, van Doornen LJ, de Visser DC, Orlebeke JF (1990). Existing and training induced differences in aerobic fitness: Their relationship to physiological response patterns during different types of stress. Psychophysiology.

[52] Spalding TW, Lyon LA, Steel DH, Hatfield BD (2004). Aerobic exercise training and cardiovascular reactivity to psychological stress in sedentary young normotensive men and women. Psychophysiology.

[53] Albright CL, King AC, Barr Taylor C, Haskell WL (1992). Effect of a six-month aerobic exercise training program on cardiovascular responsivity in healthy middle-aged adults. J. Psychosom. Res..

[54] Heydari M, Boutcher YN, Boutcher SH (2013). The effects of high-intensity intermittent exercise training on cardiovascular response to mental and physical challenge. Int. J. Psychophysiol..

[55] Badrov MB, Horton S, Millar PJ, McGowan CL (2013). Cardiovascular stress reactivity tasks successfully predict the hypotensive response of isometric handgrip training in hypertensives. Psychophysiology.

[56] Amigo I, GonzÁlez A, Herrera J (1997). Comparison of physical exercise and muscle relaxation training in the treatment of mild essential hypertension. Stress Med..

[57] Iqbal TM, Singhal K, Pal AK, Karim SM (2022). Effect of endurance training on sympathetic reactivity by sustained hand grip test. Natl. J. Physiol. Pharm. Pharmacol..

[58] Blumenthal JA (1991). Stress reactivity and exercise training in premenopausal and postmenopausal women. Heal. Psychol. Off. J. Div. Health Psychol. Am. Psychol. Assoc..

[59] Arvidson E, Dahlman AS, Börjesson M, Gullstrand L, Jonsdottir IH (2020). The effects of exercise training on hypothalamic-pituitary-adrenal axis reactivity and autonomic response to acute stress—A randomized controlled study. Trials.

[60] Throne LC, Bartholomew JB, Craig J, Farrar RP (2000). Stress reactivity in fire fighters: An exercise intervention. Int. J. Stress Manag..

[61] Goya TT (2021). Exercise training reduces sympathetic nerve activity and improves executive performance in individuals with obstructive sleep apnea. Clinics (Sao Paulo).

[62] Gupta SS, Sawane MV (2016). Effects of yoga and endurance exercise on some neurologic functions, a comparative study. Indian J. Physiol. Pharmacol..

[63] Chambers, D. *et al.* Proceedings of the 8th annual conference on the science of dissemination and implementation : Washington, DC, USA, 14–15 December 2015. *Implement. Sci.* **11**(Suppl 2), 100 (2016). 10.1186/s13012-016-0452-0 PMC4977475 27490260

[64] De Sousa EC (2017). Resistance training alone reduces systolic and diastolic blood pressure in prehypertensive and hypertensive individuals: Meta-analysis. Hypertens. Res..

[65] Smart NA (2015). Validation of a new tool for the assessment of study quality and reporting in exercise training studies. Int. J. Evid. Based. Healthc..

[66] Armijo-Olivo S (2017). Blinding in physical therapy trials and its association with treatment effects. Am. J. Phys. Med. Rehabil..

[67] Adams, S. C. *et al.* Comparing the reporting and conduct quality of exercise and pharmacological randomised controlled trials: A systematic review. *BMJ Open* **11**, 48218 (2021). 10.1136/bmjopen-2020-048218 PMC8359527 34380726

[68] Kario K (2023). Peak home blood pressure as an earlier and strong novel risk factor for stroke: The practitioner-based nationwide J-HOP study extended. Hypertens. Res..

[69] Kario K (2023). Evidence for the surge blood pressure resonance hypothesis as a trigger for cardiovascular disease events. Hypertens. Res..

[70] Lafleche AB, Pannier BM, Laloux B, Safar ME (1998). Arterial response during cold pressor test in borderline hypertension. Am. J. Physiol. Circ. Physiol..

[71] Tsai PS, Yucha CB, Nichols WW, Yarandi H (2003). Hemodynamics and arterial properties in response to mental stress in individuals with mild hypertension. Psychosom. Med..

[72] Jerusalem, M. & Schwarzer, R. Self-efficacy as a resource factor in stress appraisal processes. in *Self-Efficacy Thought Control Action*. Vol. 195213 (1992).

[73] Somani Y (2017). Reductions in ambulatory blood pressure in young normotensive men and women after isometric resistance training and its relationship with cardiovascular reactivity. Blood Press. Monit..

[74] Eisenberger NI, Taylor SE, Gable SL, Hilmert CJ, Lieberman MD (2007). Neural pathways link social support to attenuated neuroendocrine stress responses. Neuroimage.

[75] Mariano, I. M. *et al.* Different cardiovascular responses to exercise training in hypertensive women receiving β-blockers or angiotensin receptor blockers: A pilot study. *Clin. Exp. Hypertens.* (2022). 10.1080/10641963.2022.2065290. 10.1080/10641963.2022.2065290 35465803

[76] Hansen D (2018). Exercise prescription in patients with different combinations of cardiovascular disease risk factors: A consensus statement from the EXPERT working group. Sport. Med..

[77] Naci, H. *et al.* How does exercise treatment compare with antihypertensive medications ? A network meta-analysis of 391 randomised controlled trials assessing exercise and medication effects on systolic blood pressure. *Br. J. Sports Med.* (2018). 10.1136/bjsports-2018-099921. 10.1136/bjsports-2018-099921 30563873

[78] Gonçalves LF (2022). Mat Pilates training and blood pressure reactivity responses to psychological stress: Comparison between normotensive and hypertensive postmenopausal women. Blood Press. Monit..

